# Current Treatment Approaches and Outcomes in the Management of Rectal Cancer Above the Age of 80

**DOI:** 10.3390/curroncol28020132

**Published:** 2021-03-30

**Authors:** Ali P. Mourad, Marie Shella De Robles, Soni Putnis, Robert D.R. Winn

**Affiliations:** Department of Surgery, The Wollongong Hospital, Wollongong, NSW 2500, Australia; shella.derobles@gmail.com (M.S.D.R.); soni.putnis@health.nsw.gov.au (S.P.); winnro251@gmail.com (R.D.R.W.)

**Keywords:** rectal neoplasms, aged, radiotherapy, chemotherapy, surgery, survival

## Abstract

Background: The number of cases of rectal cancer in our older cohort is expected to rise with our ageing population. In this study, we analysed patterns in treatment and the long-term outcomes of patients older than 80 years with rectal cancer across a health district. Methods: All cases of rectal cancer managed at the Illawarra Cancer Care Centre, Australia between 2006 and 2018 were analysed from a prospectively maintained database. Patients were stratified into three age groups: ≤65 years, 66–79 years and ≥80 years of age. The clinicopathological characteristics, operative and non-operative treatment approach and survival outcomes of the three groups were compared. Results: Six hundred and ninety-nine patients with rectal cancer were managed, of which 118 (17%) were aged 80 and above. Patients above 80 were less likely to undergo surgery (71% vs. 90%, *p* < 0.001) or receive adjuvant/neoadjuvant chemoradiotherapy (*p* < 0.05). Of those that underwent surgical resection, their tumours were on average larger (36.5 vs. 31.5 mm, *p* = 0.019) and 18 mm closer the anal verge (*p* = 0.001). On Kaplan–Meier analysis, those above 80 had poorer cancer-specific survival when compared to their younger counterparts (*p* = 0.032), but this difference was no longer apparent after the first year (*p* = 0.381). Conclusion: Patients above the age of 80 with rectal cancer exhibit poorer cancer-specific survival, which is accounted for in the first year after diagnosis. Priority should be made to optimise care during this period. There is a need for further research to establish the role of chemoradiotherapy in this population, which appears to be underutilised.

## 1. Introduction

Colorectal cancer (CRC) is a significant international disease burden as the third most common cancer and the second leading cause of cancer-related deaths globally [1]. Owing to advancements in endoscopic screening programs together with improvements in life expectancy, the number of new cases is expected to rise to an estimated 2.2 million cases internationally by 2030 [2]. In the absence of extensive metastatic disease, surgery remains the mainstay of treatment with or without adjunctive therapy depending on the clinicopathological stage. Rectal cancers particularly may be treated with adjuvant or neoadjuvant chemoradiotherapy complementing total mesorectal excision [3]. While international guidelines provide a framework to direct the treatment approach, a recent paradigm shift has occurred whereby therapeutic options are individualised according the consensus of the multidisciplinary team (MDT) [4,5]. Factors such as age, patient preference and predicted tolerability of therapy are taken into consideration.

Our population is aging, and with this comes a rise in the number of colorectal cancer cases in older patients. Age in itself is a risk factor for CRC due to the cumulative effects of DNA damage throughout a lifetime [6]. Today, the majority of cases are diagnosed in patients above the age of 70 and an estimated 40% are above the age of 75 [3,7]. In Australia, the absolute number of patients above the age of 80 with CRC has more than doubled between 1990 and 2019, such that it is now the most prevalent cancer in this age group [8]. Treatment in this population is complicated by the presence of systemic comorbidities alongside reduced physiological reserve [9,10]. These factors may deter clinicians from offering therapeutic options that would traditionally be given to their younger counterparts due to concerns over the perceived tolerability of surgery or adjuvant treatment. This dilemma is made more complex by the fact that randomized controlled trials in CRC frequently exclude older patients from their interventions [5].

Despite this hesitancy, there has been recent data supporting the fact that older patients should not be deprived of treatment options purely on the basis of age [11,12]. Surgery in particular was previously reserved for the young as a result of unfavourable perioperative mortality rates in older patients. As perioperative care improved, a shift in practice has also occurred whereby more elderly patients with CRC are now being offered surgery alongside adjunctive treatment options [3,13]. The majority of these analyses however are retrospective in nature, and are thus subject to major limitations including large amounts of missing data and selection bias [14,15,16]. Now more than ever with our population maturing, there is a need for prospectively collected data that examines the patterns of treatment in our older patients with CRC. More importantly, it is important to explore whether the current practice complements the pathological features of cancers in this population and results in favourable outcomes.

In this study, we aimed to analyse the clinical differences, treatment approaches, pathological features and outcomes in older patients with rectal cancer, when compared with younger patients across a health district. We explored rectal cancer in particular as this subset of CRC is disproportionately more common with increasing age [17,18]. An age cut-off of 80 was selected being well above the median age at diagnosis of 70 [9,19]. Additionally, there has been growing interest in the management of octogenarians and nonagenarians across various oncological disciplines and we looked to gain further insight into this division of oncology [20,21,22].

## 2. Materials and Methods

### 2.1. Overview

This was a retrospective analysis of a prospectively maintained database of all cases of rectal cancer managed between 1st January 2006 and 31st December 2018 at the Illawarra Cancer Care Centre, New South Wales, Australia. All patients in the database were above the age of 18 and had a histological diagnosis of rectal cancer; achieved either through endoscopic sampling or following surgical resection. Patients were included regardless of whether or not they underwent surgery, neoadjuvant or adjuvant treatment. Information was entered into the database throughout the study period using a combination of electronic medical records, pathology reports, operation reports, radiological findings, MDT discussions as well as history and examination findings during routine follow up. Patients are typically followed up by their medical or radiation oncologists at 6 to 12-monthly intervals for those in remission and more frequently for those with active disease or when there is a clinical indication.

In this analysis, the population of patients was stratified into three groups based on age at the time of diagnosis: those ≤65 years (Group A), 66–79 years (Group B) and ≥80 years of age (Group C). Statistical and descriptive comparisons were made between the three groups using the defined measures and outcomes. The study was approved by the Joint University of Wollongong (UOW) and Illawarra Shoalhaven Local Health District (ISLHD) local Human Research Ethics Committee (2020/ETH00676).

### 2.2. Measures and Outcomes

Comparisons were made between the defined age groups in three different domains. In the first domain, we compared the clinical characteristics of the entire cohort of patients between the three age groups. The distribution of symptomology, body mass index (BMI) and pre-treatment carcinoembryonic antigen (CEA) levels were compared. Eastern Cooperative Oncology Group (ECOG) performance status was determined at the time of initial medical or radiation oncology consultation. Patients cancer stage was established using the framework in the eighth edition of the American Joint Committee on Cancer (AJCC) staging manual. Tumour distance from the anal verge (FAV) was measured from the caudal extremity of the rectal mass to the anal opening established through magnetic resonance imaging (MRI) of the pelvis pre-treatment. 

The second domain explored treatment approaches looking first and foremost at the proportion of patients who underwent surgery and the type of surgery undertaken. For those that did not undergo surgery, the treatment modality consisting of palliative chemotherapy and/or radiotherapy or no treatment was specified. Patients that underwent curative surgical resections were then analysed for any pathological differences between tumours in each age group. The amount of regional lymph nodes retrieved intraoperatively and involvement of the margin of the resected specimen were used as surrogates for the robustness of surgery. Histological grade, the presence of lymphovascular invasion or perineural invasion and tumour size were determined upon evaluation by the treating pathologist. Receipt of adjuvant and neoadjuvant chemo- or radiotherapy was then compared. The specific chemoradiotherapy regimens were guided by local protocols and agreed upon by members of the MDT.

Finally, the primary outcomes were examined and included overall survival (OS), cancer-specific survival (CSS) and disease-free survival (DFS) measured throughout the follow up period. Analyses of the former two survival measures included the entire cohort of patients, whereas DFS only included patients who underwent curative treatment. OS was defined as the interval from the time of diagnosis to the time of death or censored at the last follow up if the patient was alive. CSS was defined as the interval from the time of diagnosis to the time of cancer-related death or censored at the last follow up where the patient was alive. DFS was defined as the interval from the time of curative treatment to the time of recurrence or censored at the last follow up where there was no recurrence. Recurrence was defined as the histological or radiological evidence of locoregional or distant disease following completion of curative treatment. Recurrence may be guided by but was not defined by serial CEA levels during follow up.

### 2.3. Statistical Analysis

Statistical analysis was performed using SPSS V27.0 (IBM, Armonk, New York, NY, USA). Clinical characteristics, treatment approaches, pathological features and outcomes were compared between the three age groups. Where data was incomplete or missing, statistical analyses were performed using the data that was available for that characteristic. Categorical variables were presented as frequencies and analysed using the chi-squared test. Continuous variables were summarised as means ± standard deviations (SD) and were analysed using ANOVA model. Two-sided p-values less than 0.05 were deemed to be significant. OS, CSS and DFS were plotted using the Kaplan–Meier method with the logrank test used to compare the pattern of each survival curve.

## 3. Results

A total of six hundred and ninety-nine patients with rectal cancer were managed across three different centres at the Illawarra Shoalhaven Local Health District between 2006 and 2018, of which 288 (41%) were aged 65 or less (Group A), 293 (42%) were aged 66–79 (Group B) and 118 (17%) were aged 80 or above (Group C) at the time of diagnosis. The mean age of the cohort collectively was 67.5 ± 12.5 years and 66% of patients comprised of males. Median follow up throughout the study period was 45 months. Five hundred and sixty-five (91%) patients were symptomatic at the time of presentation, usually with any combination of rectal bleeding, altered bowel pattern, pain or symptomatic iron deficiency anaemia. All but four of the 699 rectal cancers were histologically classified as adenocarcinomas and 81% of patients had no evidence of distant disease at the time of diagnosis.

### 3.1. Clinical Features

Table 1 summarises the clinical differences and similarities between the three age groups. The distribution of AJCC cancer stages was comparable between Groups A, B and C (*p* = 0.240). Unsurprisingly, patients aged 80 and above were more likely to have poorer performance statuses with 57% labelled as ECOG 0 or 1, compared 97% and 92% for those aged ≤65 and 66–79, respectively (*p* < 0.001). While baseline BMI reduced slightly with increasing age, the differences were clinically insignificant as all three age groups were within a healthy BMI range (28.7 kg/m^2^ to 26.6 kg/m^2^). Additionally, while 96% of patients aged 80 above were symptomatic on presentation, compared with 89–90% in the younger age groups, this did not reach significance. The morphology of cancers was also comparable with 99%, 100% and 98% of cancers receiving a final pathological label of adenocarcinoma in Groups A, B and C, respectively. Pre-operative CEA levels numerically increased with increasing age with mean levels of 61 ng/mL, 175 ng/mL and 193 ng/mL for those aged ≤65, 66–79 and ≥80, respectively, though once again the overall pattern was not significant (*p* = 0.609). With increasing age, tumours were an average closer to the anal verge as determined on imaging (mean = 46 mm vs. 64 mm, *p* < 0.001).

### 3.2. Treatment Patterns and Pathological Differences

Table 2 details the operative and non-operative treatment patterns of the entire cohort of rectal cancer patients within each age group. Only 71% of patients in Group C underwent surgical resection for their rectal cancers, compared with 90% in Group A and 84% in Group B (*p* < 0.001). No patients in Group C underwent pelvic exenteration surgery. For patients that did not undergo any surgery, those aged 80 and above were significantly less likely to receive palliative chemo- or radiotherapy (53% vs. 77%, *p* = 0.005).

For those patients that underwent surgery (N = 589), Table 3 summarises the pathological differences in rectal cancer across the three age groups. The mesorectal specimens of patients that underwent surgery aged 80 and above were slightly less likely to contain 12 or more lymph nodes, when compared with those aged 65 and less (78% vs. 81%, *p* = 0.049). A complete pathological resection (R0 margins) was achieved less commonly with increasing age at a rate of 94%, 95% and 88% for Groups A, B and C, respectively (*p* = 0.004). Of those that underwent an R0 resection however, the tumour distance from resection margin was not significantly different between the three groups (mean = 11.7 mm vs. 10.4 mm, vs. 8.5 mm, *p* = 0.091). Tumours were on average larger in Group C when compared with patients in Group A (mean = 36.5 mm vs. 31.5 mm, *p* = 0.019), although older patients’ tumours were less likely to be poorly differentiated (9% vs. 16% vs. 6% for Groups A, B and C, respectively, *p* = 0.019). Additionally, while rates of lymphovascular invasion were comparable, tumours in those aged 80 or above were less likely to display perineural invasion compared with those 65 and below (12% vs. 19%, *p* = 0.013).

**Figure 1:**
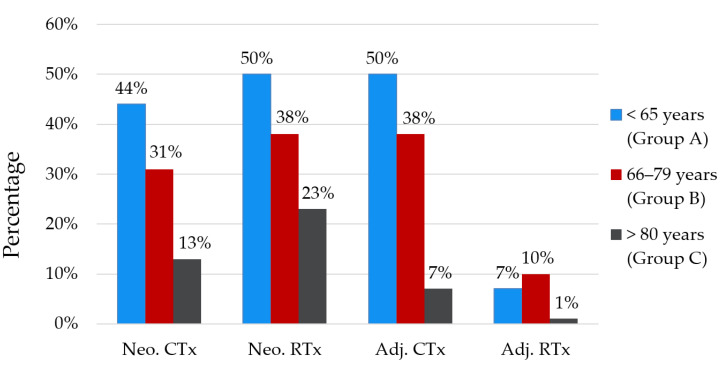
Percentage of patients with rectal cancer that received either neoadjuvant or adjuvant chemoradiotherapy concurrent with surgery. Differences between each age group within each category were significant (*p* < 0.05). Adj. = adjuvant, CTx = chemotherapy, Neo. = neoadjuvant, RTx = radiotherapy.

Figure 1 summarizes the receipt of adjuvant and neoadjuvant therapy for patients that underwent surgery for their rectal cancer, across each age group. Despite being characterised by larger tumours, closer to the anal verge with reduced R0 resection rates, patients aged 80 and above were significantly less likely to receive adjuvant or neoadjuvant chemo- or radiotherapy (*p* < 0.05 across all categories).

### 3.3. Outcomes

**Figure 2:**
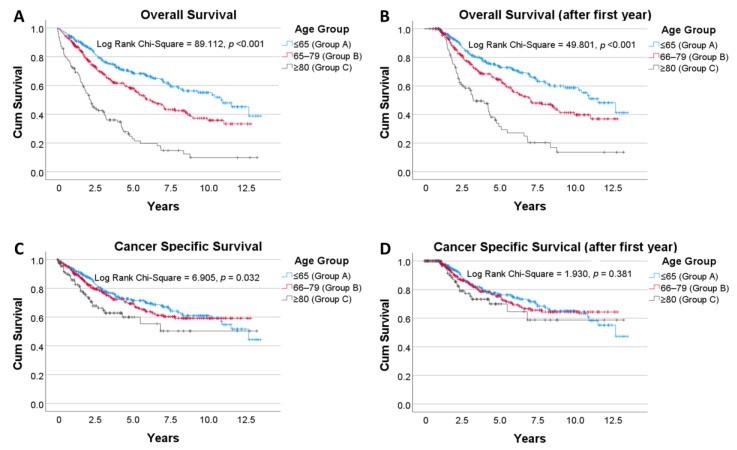
Kaplan–Meier analyses by age group. Above analyses included all patients, regardless of whether or not they underwent surgery (**A**): Overall survival. (**B**): Overall survival where deaths in the first year after diagnosis are excluded. (**C**): Cancer specific survival. (**D**): Cancer specific survival where deaths in the first year after diagnosis are excluded. Vertical tick marks along each survival curve indicate censorship—i.e., the last follow up where the patient was alive.

The median follow up period for the study’s participants was 45 months. The five-year overall mortality for the entire population of rectal cancer patients was 37.5% while the death from cancer-related causes occurred in 25.2% of patients. Figure 2 details the Kaplan–Meier survival analyses for OS and CSS for the entire cohort of rectal cancer patients. OS was significantly poorer for patients aged 80 when compared to the younger age brackets (Figure 2A), and this difference was maintained when deaths in the first year following diagnosis were excluded (Figure 2B). CSS was poorer for patients ≥80 (Figure 2C). When cancer-specific deaths in the first-year post-diagnosis were excluded, there was no significant difference in CSS between those ≤65, 66–79 and ≥80 years of age (Figure 2D).

**Figure 3:**
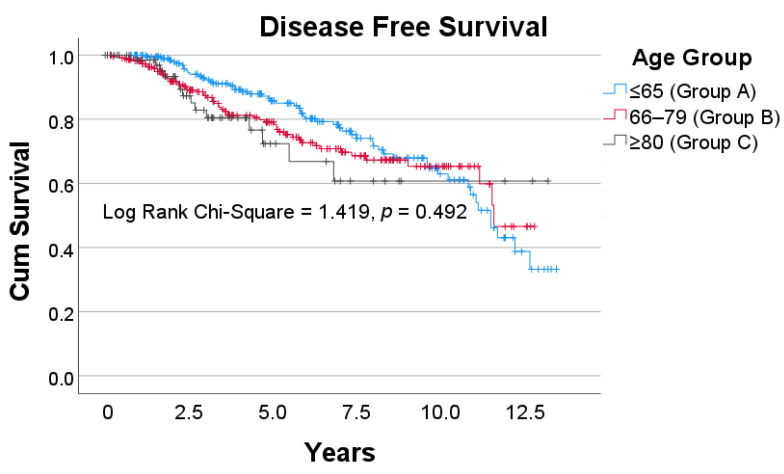
Disease Free survival for patients that underwent curative treatment across the three age groups. Vertical tick marks along each survival curve indicate censorship—i.e., the last follow up where there was no evidence of disease recurrence.

For those patients that underwent curative treatment, evidence of recurrence throughout the study period was encountered in 22.6%, 20.5% and 15.3% of patients in Groups A, B and C, respectively (*p* = 0.972). The site of recurrence was at a distant site in 79% of those aged ≤ 65, 70% in those aged 66–79 and 72% in those ≥80 years of age, with no significant difference between the three groups (*p* = 0.725). There was no difference in DFS for patients that underwent curative treatment across the three age groups (Figure 3).

## 4. Discussion

Despite growing interest, research into the management of older patients with CRC continues to lag behind the growth of this patient population [3]. Rectal cancer in particular poses additional difficulties in geriatrics. Pelvic surgery is commonly anatomically challenging, with longer operative times and larger volumes of intraoperative blood loss when compared with cancers in other areas of the colon [23,24]. This ultimately increases perioperative morbidity in a population with reduced physiological reserve. Additionally, a higher likelihood of leakage with a low colonic anastomosis frequently necessitates a diverting stoma [25]. Issues surrounding stoma care and fluid losses become increasingly pertinent, particularly if there is concurrent cardiovascular or kidney disease [5]. Through this institutional experience, we were able to explore how some of these considerations come into practice and their effects in the longer term.

In this analysis, we observed that 17% of patients with rectal cancer were aged 80 and above with a male majority, which is comparable with the demographic distribution reported in previous studies [17,26,27]. Nonetheless, we found important clinical and pathological differences between the age groups. Although not reaching significance, the average pre-treatment CEA level was more than 3-fold higher for patients ≥80 compared with those ≤60. It should be noted however that CEA levels also rise with chronic kidney disease, which is invariably more common in older patients [28]. As a result, it is difficult to determine how much of this difference is truly reflective of disease burden. For patients that underwent a surgical resection, there was a clear association between increasing age and an increase in the average size of the rectal cancer (31.5 mm vs. 36.5 mm, *p* = 0.019), although their tumours were less likely to exhibit perineural invasion and had a more favourable histological grade. In a recent study by Sell et al. exploring 1301 patients exclusively with colon cancer, patients age 80 and above similarly had larger tumours [29]. Like us, they also found that older patients tumours’ exhibited less aggressive pathological features, which may indicate there is a longer window for tumours to grow in older patients before they become symptomatic. Several multicentre studies have made clear that tumour size has a greater influence than once thought on survival in CRC compared with other components of the TNM system [30,31]. This would suggest that consideration of adjuvant treatment in older patients where feasible is ever more crucial.

Patients aged 80 and above were significantly less likely to undergo surgery compared with younger patients at our institution (71% vs. 90%, *p* < 0.005). More importantly, the nature of the surgery they underwent was less aggressive, evident through inferior R0 resection rates and the reduced proportion of specimens containing more than 12 lymph nodes. Both of these parameters are surrogates for complete oncological cure [32]. A higher fraction of positive margin specimens may in part be explained by the observation that tumours in those ≥80 are closer to the anal verge (46 mm vs. 64 mm, *p* = 0.001); alongside the competing desire to perform a sphincter preserving procedure in the elderly [33,34]. Opinions on the role of curative surgery in older patients with rectal cancer are conflicting, with some authors suggesting that a directed wait and watch program may be more appropriate [35,36,37]. An analysis of a UK national cancer registry by Birch et al. found that variation in practice was likely attributable to the heterogeneity of this population, and that careful selection of older patients for surgery results in good outcomes [38]. Several other studies mirror these results, but stress that surgical candidacy should be guided by patients’ comorbidities and frailty level rather than age [11,12,14,18,27,39].

We observed a clear trend towards a reduction in the likelihood of receiving adjuvant or neoadjuvant chemo- or radiotherapy with increasing age for patients that underwent surgery. This was despite patients ≥80 having larger and lower tumours with greater positive margin resection rates, which are factors that typically sway towards consideration of neoadjuvant and adjuvant treatment. This finding is consistent with retrospective analyses performed by Kunitake et al. and Goldvaser et al. [20,27]. Although the reasons were not reported in our database, increased toxicity together with questionable efficacy given that older patients are frequently excluded from trials appear to be the primary concerns [3,19,40]. Conversely, Choi et al. in an study of 160 patients found that pre-operative chemoradiotherapy in patients with rectal cancer above 70 resulted in comparable response rate to younger patients without increased toxicity [15]. Fiorica et al. similarly found acceptable toxicity rates in rectal cancer patients above 75 with adjuvant radiotherapy [16]. These studies however are limited by their small sample sizes and retrospective designs which result in a significant selection bias. There is a need for prospectively collected data that further explores the benefit and tolerability of chemoradiotherapy in older patients with rectal cancer, particularly in light of the inferior complete surgical excision rate in this age group.

Throughout the follow up period, patients aged 80 and above with rectal cancer had significantly poorer OS compared to their younger counterparts (*p* < 0.001). Although, age in itself is a risk factor for mortality even in the absence of disease. Thus in a study that stratifies patients based on age, OS may not be the most appropriate outcome measure. CSS was poorer for patients ≥80, but the difference was less profound (*p* = 0.032). However, no difference in CSS between the age groups was observed when first year cancer mortalities were excluded (*p* = 0.381). We performed this analysis on the basis that that previous studies reported the majority of cancer-related deaths in older patients with CRC come in the first year post-operatively [7,41,42,43,44]. We were able to replicate these findings specifically for rectal cancer. No difference in DFS was observed between the three age groups, demonstrating that acceptable remission rates were achieved in older patients undergoing curative resections.

Poorer CSS in older patients that is no longer apparent after the first year can be interpreted in two possible lights. The first is in line with our aforementioned findings, in that older patients with rectal cancer may be undertreated both with respect to surgery and neoadjuvant and adjuvant treatment. Interestingly, in a population-based study of five European countries, variations in the treatment approach between countries did not translate to a survival difference in rectal cancer patients above the age of 80 [45]. The analysis however is subject to important limitations as acknowledged by the authors, including not accounting for differences in comorbidities between nations and the exclusion of patients with missing staging data. Moreover, patients were stratified into two staging groups (I–III and IV) whereas it is known there are substantial differences in survival across substages even within each staging group [30,31]. Although opinions in the literature are conflicting, there is a respectable evidence base detailing the potential benefits of surgery and adjunctive therapy in older patients, and that modified regimens may be appropriate to mitigate any adverse effects [16,18,46,47].

Secondly, poorer CSS in the first year following diagnosis may be accounted for by perioperative mortality in the older patients. Despite recent advancements, older patients with CRC continue to demonstrate poorer perioperative outcomes when compared to their younger counterparts [43,44,48]. Risk stratification of this population pre-intervention could provide an opportunity for improvement in this facet. As mentioned, the presence of systemic comorbidities rather than age is a stronger predictor of perioperative outcomes [11,12,13,14]. Sheridan et al. in a study of more than 5000 patients with CRC found that an ASA level of III or above was an independent risk factor for 30-day mortality [6]. Several composite measures have also been developed to aid in this process. The complex geriatric assessment (CGA) is a systematic means of exploring the abilities of older patients across multiple domains and has been proven to correlate with early mortality [11,42,49,50]. Frailty indices which assess functionality through objective measures such as walk time, grip strength and muscle density can also predict the post-operative course following surgery for CRC [10,51,52].

Further to this, for those patients deemed to be high risk but appropriate candidates for surgery, attempts should be made to address any reversible risk factors for peri-operative morbidity. There are unfortunately unmodifiable factors, such as the fact that older patients with rectal cancer more frequently present in an emergency setting compared with younger patients [38]. Regarding the mode of surgery, selected studies have advocated for laparoscopic over open surgery for older patients in light of favourable short-term outcomes [53,54]. These results however are likely to be affected by bias through exclusion of patients with cardiorespiratory contraindications to pneumoperitoneum, and those with complex surgeries mandating an open procedure. A large proportion of short-term morbidity in older patients post rectal surgery can be attributed to anastomotic leakage [55]. Optimisation of nutrition and a formation of a diverting stoma in high-risk patients (e.g., those with cardiovascular disease) may be of benefit [55,56]. Finally, there is a growing evidence base detailing the benefits of prehabilitation in preparing older patients for the physiological insult of major abdominal surgery [57,58].

This study has a number of strengths, namely the inclusion of a large cohort of rectal cancer patients, which as mentioned presents its own unique challenges compared to CRC as a collective entity. Additionally, the analysis was less prone to missing data due to the prospectively maintained nature of the database. Whereas several studies only included patients that underwent surgery for their CRC, we included all managed cases of rectal cancer which offsets any selection bias with only including surgical candidates when evaluating survival outcomes. We do however acknowledge important limitations of this study. A major limitation was that our analysis did not explore the specific causes of morbidity and mortality. This may have provided further insight into the means that perioperative care for older patients can be optimised. Additionally, all cases were managed at a single health district and patterns of treatment are likely to have been influenced by departmental policies and traditions without necessarily reflecting the totality of national practice. With any long-term study, there is a dependence on reliable follow up measures. Although the last-available follow up was detailed as censorship in our survival analyses, it is likely that our survival outcomes were underreported as invariably there are patients that are lost to follow up. Finally, patients that were never referred to the district Cancer Care Centre are unlikely to have been included in the database, such as those that were actively palliated at the time of diagnosis.

## 5. Conclusions

The management of older patients with rectal cancer will only increase in importance with our ageing population. Through this institutional study, we found a number of important findings that may guide practice and research into rectal cancer above the age of 80. Older patients were less likely to receive adjuvant treatment despite larger tumours and less curative resections. Further research is needed to establish the role of chemoradiotherapy in this population under these circumstances. The difference in cancer-related mortality in older patients with rectal cancer is predominantly accounted for in the first year following diagnosis. Priority should be given to identifying those patients at greatest risk in this period and addressing any reversible risk factors under the direction of the multidisciplinary team.

## Figures and Tables

**Table 1:** Clinical characteristics of patients with rectal cancer by age group. The table here demonstrates a comparison of the entire cohort of patients, regardless of whether or not they underwent a surgical resection.

	Overall (N = 699)	Group A ≤65 Years (N = 288, 41%)	Group B 66–79 Years (N = 293, 42%)	Group C ≥80 Years (N = 118, 17%)	*p* Value
Cancer Stage					0.240
I	152 (22%)	49 (17%)	67 (23%)	36 (31%)	
IIA	91 (13%)	31 (11%)	42 (14%)	18 (15%)	
IIB	9 (1%)	4 (1%)	4 (1%)	1 (1%)	
IIC	11 (2%)	4 (1%)	5 (2%)	2 (2%)	
IIIA	50 (7%)	20 (7%)	23 (8%)	7 (6%)	
IIIB	188 (27%)	84 (29%)	75 (26%)	29 (25%)	
IIIC	64 (9%)	29 (10%)	30 (10%)	5 (4.2%)	
IVA	79 (11%)	42 (15%)	26 (9%)	11 (9%)	
IVB	55 (8%)	25 (9%)	21 (7%)	9 (8%)	
CEA (ng/mL), mean ± SD	129.4 ± 1394.1	60.8 ± 336.7	174.8 ± 1999.6	193.3 ± 1207.7	0.609
BMI (kg/m^2^), mean ± SD	28.0 ± 5.5	28.7 ± 6.2	27.7 ± 5.2	26.6 ± 4.2	0.002
ECOG ^a^					<0.001
0	243 (60%)	130 (71%)	103 (59%)	10 (20%)	
1	123 (30%)	48 (26%)	57 (33%)	18 (37%)	
2	28 (7%)	5 (3%)	12 (7%)	11 (22%)	
3	13 (3%)	1 (0.5%)	3 (2%)	9 (18%)	
4	1 (0.2%)	0	0	1 (2%)	
Presentation ^a^					0.081
Symptomatic	565 (91%)	231 (89%)	229 (90%)	105 (96%)	
Asymptomatic	58 (9%)	28 (11%)	26 (10%)	4 (4%)	
Morphology					0.543
Adenocarcinoma NOS	615 (88%)	252 (88%)	258 (88%)	105 (89%)	
Adenocarcinoma in TBA/TVA	56 (8%)	24 (8%)	24 (8%)	8 (7%)	
Adenocarcinoma mucinous	24 (3%)	10 (3%)	11 (4%)	3 (3%)	
Other	4 (1%)	2 (1%)	0	2 (2%)	
Distance FAV (mm), mean ± SD	59 ± 46	64 ± 48	59 ± 46	46 ± 40	0.001

^a^ Percentages expressed as a total of the available data for that characteristic. BMI, body mass index; CEA, carcinoembryonic antigen; ECOG, Eastern Cooperative Oncology Group; FAV, from the anal verge; NOS, not otherwise specified; SD, standard deviation; TBA, tubular adenoma; TVA, tubulovillous adenoma.

**Table 2:** Operative and non-operative treatment pattern for patients with rectal cancer across differing age groups.

	Overall	Group A ≤65 Years	Group B 66–79 Years	Group C ≥80 Years	*p* Value
Surgery ^a^					<0.001
LAR/ULAR	366 (52%)	178 (62%)	153 (52%)	35 (30%)	
APR	147 (21%)	60 (21%)	64 (22%)	23 (20%)	
Hartmann’s	29 (4%)	10 (4%)	8 (3%)	11 (9%)	
Pelvic Exenteration	4 (1%)	2 (1%)	2 (1%)	0	
Other	43 (6%)	8 (3%)	20 (7%)	15 (13%)	
No Surgery	110 (16%)	30 (10%)	46 (16%)	34 (29%)	
Treatment for those with no surgery ^b^					0.005
Palliative chemotherapy	22 (20%)	8 (27%)	13 (28%)	1 (3%)	
Palliative radiotherapy	20 (18%)	2 (7%)	8 (17%)	10 (29%)	
Palliative chemoradiotherapy	30 (37%)	13 (43%)	10 (22%)	7 (21%)	
No treatment	38 (35%)	7 (23%)	15 (33%)	16 (47%)	

^a^ Percentages expressed as a proportion of the entire cohort of patients. ^b^ Percentages expressed as a proportion of patients that underwent no surgical procedure. APR, abdominoperineal resection; LAR, low anterior resection; SD, standard deviation; ULAR, ultra-low anterior resection.

**Table 3:** Pathological differences and receipt of adjuvant/neoadjuvant therapy in rectal cancer patients that underwent surgery. Totals represent the total number of patients that underwent surgery in that age group.

	Overall (N = 589)	Group A ≤65 Years (N = 258)	Group B 66–79 Years (N = 247)	Group C ≥80 Years (N = 84)	*p* Value
Lymph Nodes retrieved ^a^					0.0498
<12	118 (21%)	48 (19%)	54 (24%)	16 (22%)	
≥12	435 (79%)	203 (81%)	176 (76%)	56 (78%)	
Resection Margin ^a^					0.004
R0	531 (94%)	239 (94%)	226 (95%)	66 (88%)	
R1	32 (5%)	15 (6%)	11 (5%)	6 (8%)	
R2	4 (1%)	0	1 (1%)	3 (4%)	
Resection Margin (mm)					0.091
mean ± SD	10.7 ± 11.5	11.7 ± 12.3	10.4 ± 11.2	8.5 ± 8.8	
Tumour size (mm), mean ± SD	34.0 ± 18.4	31.5 ± 17.4	35.9 ± 19.7	36.5 ± 16.1	0.019
Histology Grade ^a^					0.019
Well-differentiated	46 (8%)	25 (10%)	14 (5%)	7 (8%)	
Mod differentiated	292 (81%)	215 (81%)	201 (79%)	76 (86%)	
Poorly differentiated	70 (11%)	24 (9%)	41 (16%)	5 (6%)	
Lymphovascular invasion ^a^					0.058
No LVI	417 (73%)	180 (72%)	178 (74%)	59 (78%)	
LVI present	89 (16%)	33 (13%)	44 (18%)	12 (16%)	
LVI with EMVI	63 (11%)	38 (15%)	20 (8%)	5 (6%)	
Perineural invasion ^a^	77 (14%)	46 (19%)	23 (10%)	8 (12%)	0.013
Neoadjuvant Treatment ^b^					
Chemotherapy	201 (34%)	113 (44%)	77 (31%)	11 (13%)	<0.001
Radiotherapy	241 (41%)	129 (50%)	93 (38%)	19 (23%)	<0.001
Adjuvant Treatment ^b^					
Chemotherapy	229 (39%)	128 (50%)	95 (38%)	6 (7%)	<0.001
Radiotherapy	44 (7%)	19 (7%)	24 (10%)	1 (1%)	0.037

^a^ Percentages expressed as a proportion of the available data for that characteristic. ^b^ Percentages expressed as a proportion of patients that underwent surgery for their rectal cancer in that age group. EMVI, extramural vascular invasion; LVI, lymphovascular invasion; SD, standard deviation.

## Data Availability

The data presented in this study are available on request from the corresponding author. The data are not publicly available to better maintain patient privacy.
